# Pituitary cyclase‐activating polypeptide targeted treatments for the treatment of primary headache disorders

**DOI:** 10.1002/acn3.52119

**Published:** 2024-06-18

**Authors:** Nazia Karsan, Lars Edvinsson, Laszlo Vecsei, Peter J Goadsby

**Affiliations:** ^1^ Headache Group, The Wolfson Sensory, Pain and Regeneration Centre (SPaRC), NIHR King's Clinical Research Facility and SLaM Biomedical Research Centre Institute of Psychiatry, Psychology and Neuroscience, King's College London London UK; ^2^ Department of Medicine, Institute of Clinical Sciences Lund University 221 84 Lund Sweden; ^3^ Department of Neurology, Albert Szent‐Györgyi Medical School, and HUN‐REN‐SZTE Neuroscience Research Group, Hungarian Research Network University of Szeged Semmelweis u. 6 Szeged H‐6725 Hungary; ^4^ Department of Neurology University of California Los Angeles California USA

## Abstract

**Objective:**

Migraine is a complex and disabling neurological disorder. Recent years have witnessed the development and emergence of novel treatments for the condition, namely those targeting calcitonin gene‐related peptide (CGRP). However, there remains a substantial need for further treatments for those unresponsive to current therapies. Targeting pituitary adenylate cyclase‐activating polypeptide (PACAP) as a possible therapeutic strategy in the primary headache disorders has gained interest over recent years.

**Methods:**

This review will summarize what we know about PACAP to date: its expression, receptors, roles in migraine and cluster headache biology, insights gained from preclinical and clinical models of migraine, and therapeutic scope.

**Results:**

PACAP shares homology with vasoactive intestinal polypeptide (VIP) and is one of several vasoactive neuropeptides along with CGRP and VIP, which has been implicated in migraine neurobiology. PACAP is widely expressed in areas of interest in migraine pathophysiology, such as the thalamus, trigeminal nucleus caudalis, and sphenopalatine ganglion. Preclinical evidence suggests a role for PACAP in trigeminovascular sensitization, while clinical evidence shows ictal release of PACAP in migraine and intravenous infusion of PACAP triggering attacks in susceptible individuals. PACAP leads to dural vasodilatation and secondary central phenomena via its binding to different G‐protein‐coupled receptors, and intracellular downstream effects through cyclic adenosine monophosphate (cAMP) and phosphokinase C (PKC). Targeting PACAP as a therapeutic strategy in headache has been explored using monoclonal antibodies developed against PACAP and against the PAC1 receptor, with initial positive results.

**Interpretation:**

Future clinical trials hold considerable promise for a new therapeutic approach using PACAP‐targeted therapies in both migraine and cluster headache.

## Introduction to Migraine Biology

Migraine is a complex and heterogeneous brain disorder, involving dysfunction of sensory, limbic, and homeostatic regulation.^1^, ^2^ It represents the most common and disabling disorder presenting to secondary care and headache clinics worldwide, thus there is a considerable need for effective and tolerated acute and preventive therapies for its management.^3^ Widespread dynamic and oscillating changes in brain function throughout the migraine cycle^4^, ^5^, ^6^, ^7^, ^8^, ^9^, ^10^ mediate a multisymptomatic clinical phenotype involving headache, but also including symptoms such as cognitive dysfunction, sensory sensitivities, altered arousal, and mood change.^11^ Certainly, pain processing brain areas are involved in migraine, including anterior cingulate cortex (ACC), periaqueductal grey (PAG), prefrontal cortex (PFC), and thalamus.^12^, ^13^ In addition, areas distinct from other pain syndromes have been shown to be involved on functional imaging studies, including hypothalamus,^9^, ^12^, ^13^, ^14^ ventral tegmentum (VTA),^12^, ^13^ and the pons.^13^, ^15^, ^16^, ^17^ These areas are distinct from those involved in provoked cranial pain,^18^ while sharing overlap with other primary headache disorders, such as cluster headache^19^ and hemicrania continua,^20^ suggesting at some level shared neurobiology between these primary headache disorders, although certainly, of them, we understand the most about migraine thus far.

Migraine is well accepted to be a neurovascular disorder^21^; in that the interaction between sensory afferents supplying the intra‐ and extracranial vasculature, craniofacial structures, and intracranial pain processing areas, are involved in the pathophysiology. However, the drivers for migraine initiation and maintenance remain debated.^2^ It has become apparent that early symptoms preceding headache during the attack have feasible neural substrates, supporting a theory of central rather than peripheral attack initiation.^12^, ^13^, ^22^, ^23^ In addition, in those with migraine with aura, which is 20–30% of all migraine patients depending on age,^24^ the aura typically precedes headache, and aura is thought to be caused by cortical spreading depression (CSD), a wave of cortical depolarization followed by repolarization, occurring mainly over visual cortex.^25^, ^26^, ^27^, ^28^, ^29^ This may manifest as cortical hypoperfusion followed by hyperperfusion.^25^, ^28^ How, and whether this phenomenon is related to migraine headache remains unclear, as does whether CSD occurs in migraine without aura in an asymptomatic way, but clearly central changes precede headache. The persistence of brainstem imaging changes following successful abortion of headache with sumatriptan, suggests that these areas may be important to both headache initiation and maintenance.^15^

### Neuropeptides and primary headache disorders

The nociceptive innervation of the intracranial vessels and the meninges is from C‐ and Aδ fibers, primarily through the first division of the trigeminal nerve (V1), and to a lesser extent from V2 and V3, as well cervical dorsal root ganglia which supply the dura mater.^2^, ^30^ Axonal innervation of the dura mater involves several vasoactive neuropeptides, such as calcitonin gene‐related peptide (CGRP), vasoactive intestinal peptide (VIP), and pituitary adenylate cyclase‐activating polypeptide (PACAP), which are released on neuronal stimulation and cause vasodilatation.^1^, ^21^ Studies in migraine have shown that activation of perivascular nociceptive nerve fibers intra‐ and extracranially causes headache very similar to migraine, and with associated symptoms like nausea and light aversion,^31^ whereas stimulation further away from the vasculature is less nociceptive. Additionally, stimulation of dural vasculature in animals causes activation in the trigeminocervical complex (TCC) in the brainstem, an area where sensory afferent input from the trigeminal ganglion converges with that from the upper cervical cord.^32^, ^33^, ^34^, ^35^ The involvement of this area likely accounts for the typical pain distribution of migraine, involving the occipital region and neck.^36^ In cluster headache and the other trigeminal autonomic cephalalgias (TAC's), unilateral activation of the trigeminovascular pathway, with prominent activation of the parasympathetic trigeminal autonomic reflex via V1 is a key feature of the pathophysiology,^37^, ^38^ as is hypothalamic involvement.^39^ It is unclear whether activation of dural nociceptive fibers is the driver of migraine headache and if the brain changes are secondary to this, or if, perhaps, the brain changes in migraine cause a susceptibility to nociceptive pathway activation: such that usually innocuous stimuli, such as dural pulsation for example, may be perceived as painful, in the same way as unchanged light (photic hypersensitivity) and sound intensities (phonophobia) become uncomfortable during an attack. From the TCC, ascending connections to other brain areas like hypothalamus, thalamus and cerebral cortex enable pain processing and cause other migraine‐associated symptoms such as cognitive and homeostatic changes.^40^ Similarly, in the TAC's, unilateral hypothalamic involvement has been demonstrated on functional MRI studies,^19^, ^20^, ^41^, ^42^ but the link between peripheral dural nociceptor activation and central brain changes in these conditions is also unknown. Most recently evidence of premonitory symptoms occurring in cluster headache has emerged,^43^, ^44^, ^45^ as has evidence of cranial autonomic symptoms (CAS) occurring before pain onset,^43^, ^44^ both of which necessitate some reappraisal of the mechanisms involved. Taken together, the new data suggest cluster headache also involves central brain changes mediating premonitory symptoms. These are likely to occur before peripheral dural nociceptor activation, such that even activation of the parasympathetic autonomic reflex via the sphenopalatine ganglion (SPG) and superior salivatory nucleus (SSN) does not require peripheral dural afferent activation and can occur prior to headache.

### Serotonin‐based treatments for migraine

While vasodilatation may be a feature of the migraine attack, it does not seem to be significant enough to occur, at least on a level that can be captured by currently available imaging techniques, during spontaneous attacks.^46^ There is a suggestion of intracranial^38^ and extracranial vasodilatation^47^ on imaging in cluster headache. In addition, vasoconstriction for attack abortion is not necessary^48^, ^49^ nor temporally associated^50^ in both migraine and cluster headache. This understanding and the appreciation that even the triptans: serotonin 5‐hydroxytryptamine (5‐HT)_1B/1D_ receptor agonists, which are the most widely used abortive therapy in migraine, and in cluster headache along with oxygen therapy, have a largely neural basis for their action.^51^ Similarly, ergotamine, a more historic serotoninergic treatment used in the management of both disorders has a plausible neural action.^51^ Increased understanding of this biology has broadened the scope of therapeutic treatment targets in migraine and cluster headache, both within the serotoninergic system and outside. While vasodilatation mediated by vasoactive neuropeptides is a feature of both disorders, it is not the only feature and central changes are likely to be equally if not more important.^2^

A series of randomized controlled trials of lasmiditan, a specific 5‐HT_1F_ receptor agonist, for the acute treatment of migraine have demonstrated efficacy,^48^, ^49^, ^52^, ^53^ even in those with difficult to treat attacks^54^ and triptan non‐responders.^55^ These studies only identified post‐dose dizziness as an adverse effect that could limit use in some settings.^56^ Providing a novel approach, this agent does not cause vasoconstriction and therefore provides a safe treatment option for the elderly^57^ and in those with cardiovascular risk,^58^ patient groups that have historically been underserved by the triptans and by the ergot alkaloids. Lasmiditan is now US Food and Drug Administration (FDA) and European Medicines Agency (EMA) approved and is providing an option for acute treatment for many migraine patients. However, for a condition which affects more than 1 billion people worldwide, and has such global impact,^3^ there remains a paucity of targeted and effective treatments in migraine, and an ever increasing need to develop more. In particular, there is an emerging need to develop non‐vasoactive drugs to treat the disorder, given that vasoconstriction is not needed for an acute therapeutic effect, and ideally ones that do not contribute to medication overuse, which forms another challenge in the acute management of migraine.

### New treatments are still required for migraine

Understanding the many facets to migraine and primary headache neurobiology is important to allow therapeutic development strategies to be explored. As peptidergic neurotransmission at the level of the dura mater is involved in migraine and cluster headache, and results in vasodilatation and secondary central phenomena,^59^, ^60^ targeting vasoactive neuropeptides has become a substantial area of interest in migraine and cluster headache therapeutics. Recently, over three decades of research into CGRP's role in migraine as one of these neuropeptides,^61^ has led to the emergence of small molecule receptor antagonists (gepants) and monoclonal antibodies against the CGRP peptide or the receptor (mAbs) becoming part of our clinical practice and transforming the lives of many with migraine.^62^ All the phase 3 trials of the four different mAbs and seven gepants have shown consistent efficacy and good tolerability; however, these drugs are not useful for everyone and in some, may be contraindicated by active vascular disease because of theoretical concerns about inhibition of CGRP causing vasoconstriction. There is suggestion that there is an association between erenumab response and triptan response,^63^ and an increased response to triptans in mAb super‐responders,^64^ while increasing numbers of previous preventives tried negatively impacts mAb efficacy.^65^ The need for additional therapies for groups like triptan non‐responders, who may be less likely to respond to CGRP‐targeted therapies, and those with more refractory disease with several failed preventives, is therefore real. The use of treatment prediction and treatment biomarkers is an area in migraine that has been disappointing thus far, so identifying targets with distinct downstream intracellular signaling mechanisms that lack vasoactive effects is crucial to advance therapeutics in this area.

One such strategy that has been explored has been targeting PACAP.^66^

## Pituitary Cyclase‐Activating Polypeptide 38 (PACAP38)

### Structure, function, and distribution in the nervous system

PACAP^67^ belongs to the vasoactive intestinal polypeptide (VIP)–secretin–growth hormone‐releasing hormone–glucagon superfamily,^68^, ^69^ and is found as a 38‐amino acid peptide (PACAP38) and a truncated 27‐amino‐acid peptide (PACAP27).^70^ PACAP shares 68% of its amino acid homology with VIP and therefore these two peptides share four receptors: PAC_1_, VPAC_1_, VPAC_2_, and a Mas‐related G‐protein‐coupled receptor (member B2 in mouse and X2 in humans).^71^

PACAP38 is the predominant mammalian peptide and represents more than 90% of the total PACAP content in most tissues, including the central nervous system (CNS)^68^; but both isoforms have similar functions and receptor binding affinity.^71^ PACAP was first identified in bovine hypothalamus in 1989. It was found to localize in sensory neurons^72^ and could depress C‐fiber‐evoked responses.^73^ Furthermore, it was demonstrated that PACAP extract could stimulate anterior pituitary cells in rats.^74^ PACAP was subsequently found to be involved in many physiological and endocrinological functions, including vasodilatation,^75^, ^76^ and circadian rhythm^77^ and feeding regulation.^78^

### PACAP expression

**Figure 1:**
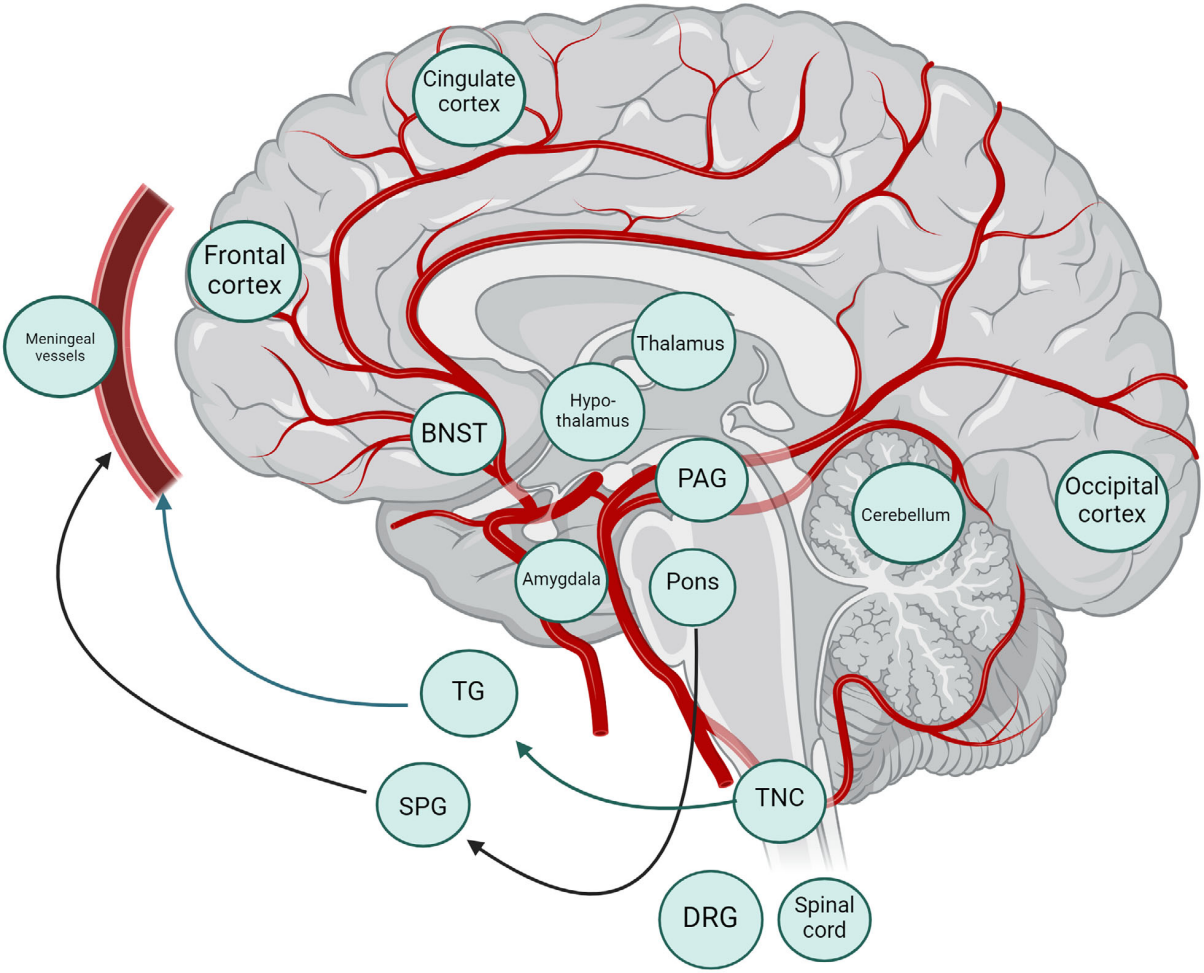
Summary of the main expression sites of PACAP and its receptors within the brain, cranial and spinal ganglia, spinal cord, and extracranial vasculature. DRG; dorsal root ganglion, PAG; periaqueductal grey, SPG; sphenopalatine ganglion, TG; trigeminal ganglion, TNC; trigeminal nucleus caudalis. Image created using BioRender.com.

Peripherally, PACAP is expressed in several cranial ganglia relevant to migraine and cluster headache biology, including the sphenopalatine ganglion (SPG)^79^, ^80^, ^81^, ^82^ and trigeminal ganglion,^83^, ^84^ which have been examined the most closely with regards to co‐expression of CGRP and PACAP.^85^, ^86^ PACAP mRNA for three PACAP receptors is found in middle meningeal artery.^87^ Whereas CGRP is widely expressed on trigeminal neurons within the trigeminal ganglion, PACAP38 is found in far fewer trigeminal neurons,^72^, ^81^ but is more widely distributed in parasympathetic neurons, leading to interest in the involvement of this peptide in both migraine and the TACs; the latter include prominent cranial autonomic symptoms in their canonical phenotypes. VIP has been historically been implicated in the biology of cluster headache, because of its role in mediating cranial autonomic symptoms, and a study suggesting its release during spontaneous cluster attacks.^88^ It has been hypothesized that via PACAP stimulating trigeminal CGRP release, the sensory and autonomic systems interact in migraine and cluster headache biology.^89^, ^90^ Centrally, PACAP and its receptors, like CGRP, are also widely expressed in brain areas of interest in migraine, such as thalamus, locus coeruleus, parabrachial nucleus and TNC. Recent work showed that PACAP38 had a weak expression with CGRP in trigeminal neurons; experiments showed that PACAP38 was only released from the trigeminal neurons and not the trigeminal nerve fibers and that its receptors were observed only on the adjacent satellite glial cells,^91^ suggesting a somewhat distinct role in pain processing as compared to that of CGRP. A summary of the main areas of PACAP expression of interest in the primary headache disorders is shown in Figure 1.

### PACAP receptors

**Figure 2:**
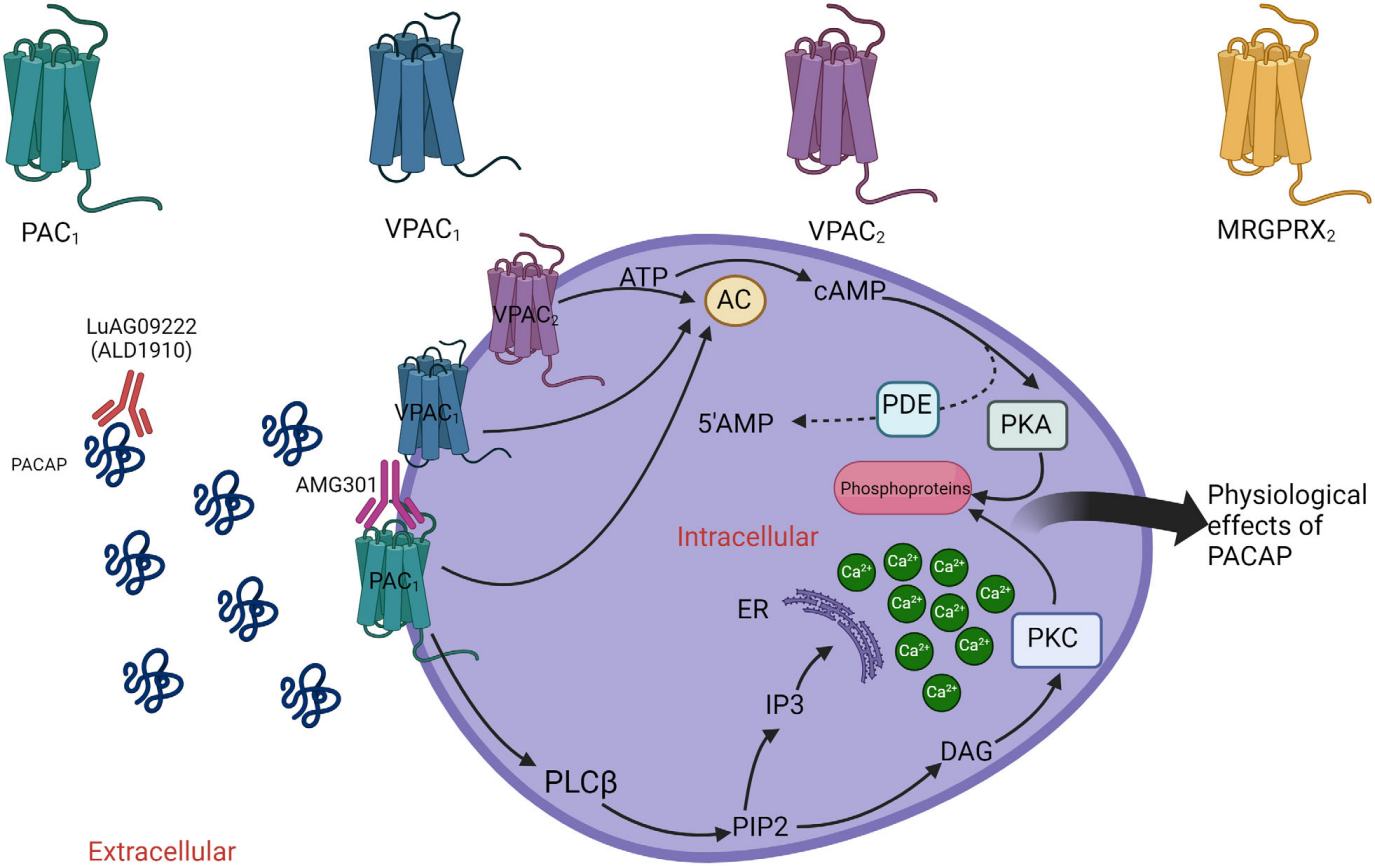
Summary of the intracellular downstream effects of PACAP. PACAP binds to four different G‐protein‐coupled receptors (PAC_1_, VPAC_1_, VPAC_2_, and MRGPRX2) to exert its effects. Receptor activation of PAC_1_, VPAC_1_, and VPAC_2_ causes release of intracellular cyclic adenosine monophosphate (cAMP) from adenosine triphosphate (ATP) via adenylate cyclase (AC). cAMP is degraded to 5′adenosine monophosphate (5′7AMP) via phosphodiesterases (PDE). cAMP causes protein kinase A (PKA) activation and phosphoprotein production. Activation of PAC_1_ also causes release phopsholipase Cß (PLC), which hydrolyzes phosphatidylinostiol 4,5‐biphosphate (PIP2) and produces intracellular mediators inositol triphosphate (IP3) and diacylglycerol (DAG), which lead to increased intracellular calcium (Ca^2+^) release from the endoplasmic reticulum (ER) and phosphoprotein production via protein kinase C (PKC), respectively. These are responsible for the physiological effects of PACAP, including trigeminal neuronal sensitization. ALD1910 and AMG301 are two mAbs developed for migraine treatment. Lu AG09222 (ALD1910) binds to the PACAP ligand, and AMG301 binds to the PAC_1_ receptor. Both hold therapeutic scope for migraine. Image created using BioRender.com.

PACAP mediates its actions via three G‐protein‐coupled receptors: VPAC_1_ and VPAC_2_ receptors, for which PACAP and VIP have equal affinity, and the PAC_1_ receptor, which is about 100‐fold more PACAP specific.^92^ Receptor activation causes cAMP release, which is among one of the mechanisms responsible for the intracellular and physiological effects of PACAP38. The PAC_1_ receptor also stimulates intracellular calcium and protein kinase C activation.^93^ Experimental animal models have shown that elevated cAMP can sensitize trigeminal neurons.^94^ In addition, recent evidence has shown PACAP38 can activate the meningeal mast cells through Mas‐related G‐protein coupled receptor member B2 (MrgprB2) and promote pain behavior in mouse through the mouse homologue receptor to the human MRGPRX2 receptor,^67^ so this has emerged as a fourth PACAP receptor of interest. See Figure 2 for a summary of the four PACAP receptors, and their downstream intracellular signaling mechanisms by which receptor activation causes physiological effects of PACAP.

Each of the PAC_1_, VPAC_1_, and VPAC_2_ receptors and their mRNA are expressed on human cerebral arteries^87^ and cranial sensory and autonomic ganglia.^95^ In an experimental migraine model, superior sagittal sinus stimulation causes PACAP release into the extracerebral circulation, where it mediates vasodilatation.^96^, ^97^ Interestingly, this is only reversed by VPAC_1_ antagonism, suggesting that PAC_1_ agonism does not contribute to vasodilatation.^98^ In another study, both VIP and PACAP‐38 caused short‐lived meningeal vasodilation mediated by the VPAC_2_ receptor independently of activation of central trigeminovascular neurons. Intravenous delivery of a PAC1 receptor antagonist specifically inhibited the meningeal vasodilatory effects of dural trigeminovascular activation, whereas only central (intracerebroventricular) administration of the drug inhibited dural nociceptive‐evoked action potentials in central trigeminovascular neurons, suggesting that a central role of PACAP in migraine is likely in addition to the primary afferent innervation in the meninges.^99^ In another animal model, middle meningeal artery dilatation was only inhibited via the PAC_1_ receptor, and not via VPAC_1_ or VPAC_2_,^87^ again suggesting that PAC_1_ may be a target without vasoconstrictive effects. Notably, in the sphenopalatine ganglion only the VPAC_1_ receptor was found and not VPAC_2_ or PAC_1_,^95^ which has led to interest in this receptor in particular in migraine and cluster headache therapy.

## PACAP38—Preclinical Evidence in Migraine and Cluster Headache

The PAC_1_ receptor is more specific to PACAP compared to VIP, and has therefore had the most interest in migraine,^100^ particularly given initial suggestions that VIP was not implicated in the pain part of migraine,^101^ although there are suggestions of its role in inducing episodic cluster headache in bout when administered intravenously.^102^ PACAP and VIP, as well as being from the same neuropeptide family and acting on similar receptors, also functionally interact. PACAP fibers innervate VIP neurons^103^ and PACAP promotes VIP gene expression^104^ and VIP release,^105^ suggesting a possible role of both peptides in both migraine and cluster headache.

Unlike CGRP, which is a somewhat larger molecule peptide and is thought to not penetrate the blood–brain barrier in significant concentrations, PACAP has a transporter pump offering the ability to cross the blood–brain barrier.^106^ This may explain why premonitory symptoms are less readily clinically provoked by CGRP compared to NTG and PACAP.^107^, ^108^, ^109^, ^110^ PACAP is heavily expressed in the hypothalamus,^71^ and this area of the brain is thought to be crucial in mediating premonitory symptoms.^8^, ^13^, ^111^ Moreover, these symptoms have also been more recently been identified as occurring in cluster headache.^12^, ^43^, ^44^, ^45^ Interestingly, PACAP is only released in the trigeminal ganglion and not from the sensory nerve fibers or in the dura mater of rat.^91^ PACAP receptors: PAC_1_ and VPAC_1/2_, are found on trigeminal ganglion neurons and satellite glial cells.^112^ Taken together, these data suggest a potential therapeutic target against PACAP may need to include the central nervous system to some extent.^85^, ^99^ Germane to this, post‐traumatic stress disorder is associated with PACAP, particularly via PAC_1_‐mediated mechanisms.^113^, ^114^

### PACAP and the trigeminovascular system

In animal models, PACAP has a role in trigeminovascular sensitization and photophobia.^115^ PACAP and VIP both cause vasodilatation,^87^ but only PACAP causes TCC activation and hypersensitivity to somatosensory cranial stimulation.^99^ When PACAP is locally applied to meningeal afferents, it promotes the central release of CGRP from the TNC, whereas VIP has no effect.^89^ In line with these observations, PACAP38‐induced migraine‐like attacks do not involve changes in blood CGRP levels.^116^ When complete Freund's adjuvant is applied to rat dura mater or orofacially to provoke inflammation, tissue necrosis, and ulceration, PACAP immunoreactivity was increased in the TNC.^117^, ^118^ PACAP also causes mast cell degranulation, which may contribute to its effect in migraine.^119^ PACAP is elevated in the TNC in rat after nitroglycerin (NTG) injection,^120^ and NTG is a commonly used migraine trigger in experimental study of migraine in humans and in animal models of migraine.

### PACAP centrally

NTG promotes PACAP immunoreactivity in the TNC in a rodent model,^115^, ^120^ and nitric oxide mechanisms may therefore play a role in neuronal sensitization via PACAP in migraine. Unlike CGRP which is pro‐nociceptive, PACAP has pro‐ and antinociceptive properties and can reduce TCC responses to dural stimulation when locally injected into the VTA parabrachial pigmented nucleus.^121^ PACAP‐deficient mice exhibit increased thermal allodynia (antinociceptive), but centrally PACAP mediates neuronal excitation in areas like PAG and somatosensory cortex (pro‐nociceptive), so there are divergent effects peripherally and centrally.^122^, ^123^ Central excitatory effects of PACAP have been demonstrated in other studies, via areas of interest in migraine such as the hypothalamus,^124^ and the TCC.^99^ A rodent study of nociceptive trigeminovascular activation demonstrated that PACAP causes activation of trigeminocervical neurons with sensitization and that this effect is reversed only through a centrally administered PAC_1_ receptor antagonist. The same drug administered intravenously reduced the peripheral result of central trigeminovascular activation, with reduced meningeal vasodilatation. This study, therefore, suggested that a central PAC_1_ mechanism is important in migraine and could be exploited for clinical therapeutics.^99^ PACAP may also have a modulatory role in facilitating trigeminovascular nociception at the level of the hypothalamus via a PAC_1_ mechanism.^124^ In addition, kynurenic acid^125^ inhibits increased expression of PACAP in the trigeminal nucleus caudalis evoked by trigeminal ganglion stimulation,^126^ suggesting that PACAP may be mediating its role in migraine via glutamatergic neurotransmission.

### PACAP, photophobia, and allodynia

A shared role of CGRP and PACAP in migraine biology is in mediating photophobia. However, these mechanisms are likely to be distinct, given that both can trigger light aversive behaviors in animal models, but CGRP‐mediated light aversion could only be inhibited by a CGRP monoclonal antibody and not a PACAP one, and vice versa.^127^ PACAP can also provoke periorbital allodynia in animal models, and this can be reversed with a PACAP receptor antagonist.^128^ Other mouse models have also suggested distinct roles of CGRP and PACAP in mediating migraine‐like behaviors, such as hypersensitivity as a correlate for allodynia.^129^ Mice pretreated with an anti‐CGRP antibody and receptor activity‐modifying protein 1 (RAMP‐1) knockout mice lacking CGRP receptors can still display allodynic behaviors in response to PACAP, but NTG‐mediated allodynia could be prevented by anti‐CGRP antibodies and in the RAMP‐1 knockout mice. NTG and CGRP, therefore, are likely to act via shared mechanisms and these are distinct from PACAP in mediating sensory sensitivities, but the interactions of these molecules in the wider migraine phenotype remain unclear. A role of PACAP in intracellular signaling pathways, some shared with CGRP given the co‐expression of both peptides in many brain regions involved in migraine, like those via cAMP, is also likely.^130^

## PACAP38—Clinical Evidence in Migraine and Cluster Headache

### PACAP is released in migraine and cluster headache

PACAP, like CGRP, is released during acute migraine in both experimental and clinical settings, and blood levels are reduced following sumatriptan administration.^96^ PACAP is also released into the circulation during episodic cluster headache bouts compared to the interbout period.^131^ There is also suggestion that blood levels of PACAP fluctuate in a dynamic fashion during different phases of migraine and may actually be lower in patients with migraine compared to healthy controls interictally, but peak ictally.^132^ Interestingly, PACAP levels are elevated in migraine attacks, but VIP levels are only elevated if there are cranial autonomic sysmptoms associated,^59^ and both are elevated in the cranial circulation in cluster headache,^88^, ^131^ a condition in which CAS are necessary for diagnosis. This suggests that PACAP and VIP are both involved in the mediation of CAS, with PACAP having additional roles in trigeminovascular nociceptive processing. Inter‐attack PACAP levels in migraine patients correlate with mean diffusivity in white matter,^133^ so these may serve a biomarker role as therapeutic avenues are explored.

### PACAP administration can trigger migraine and cluster headache attacks

PACAP38 can also trigger migraine‐like attacks,^134^ cluster headache attacks,^102^, ^135^ and premonitory symptoms associated with migraine^107^ when infused intravenously into patients with migraine, cluster headache in bout or chronic cluster headache. PACAP38 triggers migraine when infused more readily than VIP in a double‐blind crossover head‐to‐head study (73% vs. 18%).^136^ More recently, a prolonged VIP infusion has been shown to provoke migraine attacks in 71% in one study (compared to 5% with placebo),^137^ and VIP infusion was associated with prolonged extracranial vasodilatation.^138^ An earlier study had suggested no difference in headache reporting following VIP and placebo, despite marked extracranial vasodilatation after VIP.^101^ PACAP and VIP triggering rates in episodic cluster headache in bout and in chronic cluster headaches were equivalent between the two peptides in one study.^102^ VIP‐targeted treatments have thus far not been explored in migraine or cluster headache.

Interestingly, PACAP38 infusion is not associated with regional cerebral blood flow changes in human healthy volunteers,^139^ but PACAP‐triggered migraine can be prevented with pre‐.medication with sumatriptan.^140^ Angiographic imaging has shown that like NTG and CGRP, PACAP is a vasodilator of the extracranial vasculature and does not affect intracranial arteries.^136^, ^141^, ^142^ PACAP‐triggered migraine is associated with widespread functional brain network changes.^143^ There is a suggestion that PACAP27 can also trigger migraine,^144^ and cause extracranial vasodilatation.^145^

A summary of the similarities and differences between CGRP and PACAP receptors, expression and mechanisms in migraine is shown in Table 1.

**Table 1:** A summary of some of the similarities and differences between CGRP and PACAP in migraine biology (developed using^129^).

	CGRP	PACAP
Receptors (all G‐protein‐coupled)	Canonical CGRP receptor (GPCR calcitonin receptor‐like receptor CLR and RAMP1) AMY_1_ with equal affinity Lower affinity for CLR/RAMP2 and CLR/RAMP3 adrenomedullin receptors	PAC_1_ VPAC_1_ VPAC_2_ MRGPRX2
Migraine mechanisms	Vasodilation, dural mast cell degranulation, pro‐nociceptive	Vasodilation, dural mast cell degranulation, peripherally antinociceptive, centrally nociceptive
Infusion triggers migraine‐like headache	63%	72%
Infusion triggers premonitory symptoms before migraine‐like headache	9%	48%
Vasodilatory side effects of infusion (flushing, feeling hot, palpitations, and light‐headedness)	Yes	Yes
Nervous system distribution	Shared other than CGRP more in trigeminal ganglion	Shared other than PACAP more in sphenopalatine ganglion
Role in anxiety behaviours	Yes	Yes
Role in light aversion	Yes	Yes (mechanism distinct to CGRP)
Role in allodynia	Yes	Yes (although perhaps via mechanisms distinct to CGRP/nitric oxide)
Intracellular mechanisms	Less robust intracellular mechanisms via inositol 1,4,5‐triphosphate	More robust intracellular mechanisms via inositol 1,4,5‐triphosphate (via non‐canonical cAMP mechanisms and exchange protein directly activated by cAMP (EPAC's)
Action on ATP‐sensitive potassium channels	Yes	Only partial effect

## PACAP—Therapeutic Scope in Migraine

### Targeting the PAC1 receptor

A rodent‐specific PAC_1_ receptor antibody reduced TCC firing in response to stimulation, mediated via its binding at the trigeminal and sphenopalatine ganglia, without central binding.^146^ This study further supported the role of targeting the PAC_1_ receptor in migraine therapeutics. A subsequent phase 2 clinical study unfortunately showed that a PAC_1_ receptor antibody: AMG 301, was ineffective in migraine prevention at different doses.^147^

A preclinical study used a PAC_1_ receptor antibody to map PAC_1_ receptor expression in the trigeminal‐autonomic system in both rat and man using immunohistochemistry and in situ hybridization.^81^ The study demonstrated PAC_1_ immunoreactivity in SPG with only weak expression in trigeminal ganglion, and strong immunoreactivity in spinal trigeminal nucleus, but no expression in dura mater vessels, and these findings were confirmed with in situ hybridization.^81^ This finding was consistent with previous data and suggests that the vascular effects of PACAP are mediated by the VPAC receptors rather than the PAC_1_ receptor,^95^ making targeting PAC_1_ an exciting strategy for migraine potentially without substantial vascular effects, akin to specific targeting of the 5HT_1F_ receptor in the serotoninergic system.

The disappointing lack of success of a PAC_1_ receptor antibody led to the development of strategies to target the PACAP ligand, and also raised the possibility of targeting VPAC_1/2_ receptors as an approach in migraine. This work has also led to consideration of the concept that PACAP may be mediating its actions in migraine, such as direct actions at the TNC, to stimulate CGRP release where PACAP and CGRP are co‐localized,^89^ or via its effect on mast cells,^67^ or at alternative yet‐to‐be identified receptors.

Recently, PAC_1_ small molecule antagonists have been identified, one of which seems to show anti‐allodynic effects in an animal neuropathic pain model when administered orally or intrathecally,^148^ and another showing anti‐nociceptive effects in a rodent inflammatory pain model.^149^ Interestingly, a role of these agents in anxiety behaviors has also been identified.^150^ Whether these are tested in migraine models remains to be seen.

### Targeting PACAP directly

Lu AG09222 (ALD1910) is a high‐affinity PACAP‐neutralizing antibody which binds the ligand. A phase 1 double‐blind parallel‐group placebo‐controlled study showed it could inhibit cephalic vasodilatation induced by PACAP in healthy volunteers, as well as reduce PACAP‐ and VIP‐induced facial vasodilatation and mild headache.^151^ Subsequently, a phase 2 double‐blind placebo‐controlled trial showed a high dose (750 mg) of Lu AG09222 compared to a low dose (100 mg) and to placebo reduced migraine frequency over a 4‐week endpoint in participants with 2–4 previous preventive treatment failures.^152^ High dose Lu AG0222 reduced monthly migraine days by −6.2 (compared to placebo −4.2), with a baseline migraine frequency across the groups of mixed episodic and chronic migraine, of 16.7 migraine days per month.^153^ This study has paved the way for further development of approaches to this target.

## PACAP—Therapeutic Scope in Cluster Headache

While no dedicated studies using PACAP‐targeted treatments have been conducted in cluster headache, PACAP targeting poses an exciting opportunity for treating cluster headache based on the known pathophysiology. Features of cluster headache include the prominent cramial autonomic symptoms are experienced by most cluster headache patients, and the equivalent triggering of cluster headache attacks by VIP and PACAP38, which share receptors, as well as the shared neurobiology between migraine and cluster headache suggest that targeting PACAP may hold promise in cluster headache in the future. If phase 3 studies with Lu AG09222 are successful in migraine, this could lead to dedicated cluster headache trials, for a population of patients who have very few available treatment options.

## The Need for More

While PACAP poses an exciting therapeutic avenue in migraine and cluster headache, thus far we only have a failed phase 2 trial of a PAC_1_ receptor antibody and an encouraging phase 2 trial of a ligand PACAP antibody with results available. Given emerging evidence that VIP may have a role in migraine induction, based on the more recent triggering data,^137^ and demonstration of elevated blood levels interictally in migraine,^154^ as well as ictally in both blood and saliva,^155^ contrary to what had been thought previously, further targeting of VPAC_1/2_ receptors is likely to be an avenue of future interest. While treatment prediction and biomarkers of treatment response are not currently available, the emergence of targeted peptidergic therapies and the ability of these peptides to trigger migraine and cluster attacks in some patients may allow us to witness a time where triggering efficacy allows treatment efficacy to be estimated. This would ideally lead to personalization of treatment with potentially high‐cost drugs.

The CGRP‐related peptides; adrenomedullin and amylin, both of which are able to provoke migraine attacks in patients via the amylin analog pramlintide (with equivalent efficacy to CGRP),^156^ and intravenous adrenomedullin,^157^ have also gained interest as possible therapeutic substrates in migraine, providing alternative receptors as targets. The CGRP‐targeted therapies used in migraine management may have weak effects at the amylin receptor AMY_1_,^158^, ^159^, ^160^ and also alter adrenomedullin signaling at the canonical CGRP receptor.^161^

As well as targeting neuropeptides, intracellular targets such as nitric oxide, and ion channel targets like potassium channels, are also of interest in advancing migraine therapeutics.^162^

## Conclusion

Targeting the PACAP pathway, as a neuropeptide pathway distinct from CGRP, holds therapeutic promise in migraine therapeutics going forwards. Further targeted treatment development against the VPAC_1/2_ receptors may yield exciting results in this area. Moreover, the presence of cranial autonomic symptoms may serve as a future biomarker for response to targeted therapeutics against the cranial parasympathetic projection, modulated via PACAP.^163^

In migraine therapeutics development, there has not yet been a false‐positive phase 2 study, so it is hopeful that PACAP‐targeted therapies will be the next big advance in migraine therapeutics, offering some mechanisms distinct from CGRP to help those underserved by these therapies. This effect may be translated to other primary headache disorders, including cluster headache and the other TAC's in due course, exploiting cranial autonomic symptoms as a potential biomarker. However, there will always remain a need for the identification of new targets to help deal with the burden that is migraine.

## AUTHOR CONTRIBUTIONS

LE, LV, and PJG were involved in conceptualization. NK, LE, LV, and PJG were involved in the literature review, analysis and interpretation of the results, and manuscript preparation.

## Acknowledgments

None.

## Funding Information

None.

## Conflict of Interest Statement

NK has nothing to declare. LE has given talks sponsored by Pfizer, Abbvie, TEVA, and Novartis. LV has nothing to declare. PJG reports, over the last 36 months, grants from Celgene and Kallyope, and personal fees from Aeon Biopharma, Abbvie, Aurene, CoolTech LLC, Dr Reddy's, Eli‐Lilly and Company, Linpharma, Lundbeck, Pfizer, PureTech Health LLC, Satsuma, Shiratronics, Teva Pharmaceuticals, Tremeau, and Vial, and personal fees for advice through Gerson Lehrman Group, Guidepoint, SAI Med Partners, Vector Metric, and fees for educational materials from CME Outfitters and WebMD, and publishing royalties or fees from Massachusetts Medical Society, Oxford University Press, UptoDate and Wolters Kluwer, and a patent magnetic stimulation for headache (No. WO2016090333 A1) assigned to eNeura without fee.
