# Correction to “Pituitary Cyclase‐Activating Polypeptide Targeted Treatments for the Treatment of Primary Headache Disorders”

**DOI:** 10.1002/acn3.70409

**Published:** 2026-04-27

N. Karsan, L. Edvinsson, L. Vecsei, and P. J. Goadsby, “Pituitary Cyclase‐Activating Polypeptide Targeted Treatments for the Treatment of Primary Headache Disorders,” *Annals of Clinical and Translational Neurology* 11, no. 7 (2024): 1654–1668, https://doi.org/10.1002/acn3.52119.

The title should read ‘Pituitary adenylate cyclase activating polypeptide targeted treatments in primary headache disorders’.

We apologize for this error.

